# Molten Salt‐Assisted Synthesis of Titanium Nitride

**DOI:** 10.1002/smtd.202400228

**Published:** 2024-06-10

**Authors:** Mahsa Parvizian, Nico Reichholf, Aysha A. Riaz, Prajna Bhatt, Anna Regoutz, Jonathan De Roo

**Affiliations:** ^1^ Department of Chemistry University of Basel CH‐4058 Basel Switzerland; ^2^ Department of Chemistry University College London 20 Gordon Street WC1H 0AJ London UK

**Keywords:** ceramics, molten salts, nitrides, solid state synthesis

## Abstract

Titanium nitride is an exciting plasmonic material, with optical properties similar to gold. However, synthesizing TiN nanocrystals is highly challenging and typically requires solid‐state reactions at very high temperatures (800–1000°C). Here, the synthesis of TiN nanocrystals is achieved at temperatures as low as 350°C, in just 1 h. The strategy comprises molten salt, Mg as reductant and Ca_3_N_2_ as nitride source. This brings TiN from the realm of solid‐state chemistry into the field of solution‐based synthesis in regular, borosilicate glassware.

## Graphical abstract

Titanium nitride is an exciting plasmonic material, with optical properties similar to gold. Here, the synthesis of TiN nanocrystals is achieved at temperatures as low as 350°C. The strategy comprises molten salt, Mg as reductant and Ca_3_N_2_ as nitride source. This brings TiN from the realm of solid‐state chemistry into the field of solution‐based synthesis.

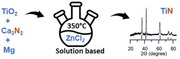

## 1 Introduction

Metal nitrides are an interesting yet underdeveloped materials class.^[^ ^1^ ^]^ Specifically, group 4 metal nitrides are characterized by a high melting point, exceptional chemical stability, and corrosion resistance. TiN, ZrN, and HfN all adopt a cubic crystal structure within the $\mathrm{Fm}\bar{3}m$ space group, the metals forming an fcc lattice and nitrogen atoms occupying the octahedral holes. Group 4 nitride nanocrystals (NCs) exhibit a localized surface plasmon resonance (LSPR) in different spectral regions. Hafnium nitride (HfN) and zirconium nitride (ZrN) display their LSPR in the visible spectrum, while for titanium nitride (TiN) it occurs in the NIR range.^[^ ^2^, ^3^ ^]^ This plasmonic response allows for the efficient conversion of sunlight into heat, benefiting applications like water evaporation and desalination,^[^ ^4^, ^5^, ^6^, ^7^, ^8^ ^]^ as well as enabling photothermal therapies and plasmon‐induced photocatalysis.^[^ ^9^ ^]^ Furthermore, ZrN NCs exhibit remarkable activity and long‐term stability, surpassing even platinum, as catalysts for the electrochemical oxygen reduction reaction.^[^ ^10^ ^]^ Similarly, HfN NCs demonstrate catalytic activity in the oxygen evolution reaction.^[^ ^11^ ^]^ However, the synthesis of these materials remains a significant challenge.

Giordano et al. pioneered the “urea glass route” to form transition metal nitrides.^[^ ^12^ ^]^ In the case of TiN, TiCl_4_ is dissolved in ethanol with urea. After evaporation, the resulting gel is heated under N_2_ at 800 °C  for 3 h. This method has been extensively used to prepare metal nitrides.^[^ ^10^, ^11^, ^13^, ^14^, ^15^ ^]^ On the other hand, there are also solid‐state approaches developed. Titanium oxide nanoparticles react with urea at 800 °C  to TiN powders.^[^ ^16^ ^]^ Recently, Dasog and coworkers synthesized plasmonic TiN, ZrN, and HfN NCs by employing Mg_3_N_2_ as the nitrogen source.^[^ ^2^ ^]^ The approach involves a solid‐state metathesis reaction at 1000 °C using commercially available TiO_2_ (17 nm), ZrO_2_ (19 nm), and HfO_2_ (43 nm).

(1) $$6\;{\mathrm{TiO}}_{2}+4\;{\mathrm{Mg}}_{3}N_{2}\overset{1000^{\circ }C}{\underset{\mathrm{Ar}\;\mathrm{flow}}{\to }}6\;\mathrm{TiN}+12\;\mathrm{MgO}+N_{2}.$$

The final powder is subjected to 1M HCl treatment to remove MgO and unreacted Mg_3_N_2_ and the final product can be dispersed in water. Transmission electron microscopy (TEM) reveals NC aggregation, and all particles show surface oxidation. This scalable approach appears effective for producing high‐quality group 4 nitrides, and other research groups have adopted it to produce plasmonic HfN NCs.^[^ ^17^ ^]^

Molten salts are solid under standard conditions, and become liquid at elevated temperatures. These salts are often the solvent of choice for solution‐based chemistry at high temperatures.^[^ ^18^, ^19^ ^]^ They are usually used as an eutectic mixture, with commonly used eutectic salts including the chloride salts (LiCl, KCl, NaCl, MgCl_2_), nitrates salts (LiNO_3_, NaNO_3_, KNO_3_), sulfate salts (Na_2_SO_4_, K_2_SO_4_), and fluoride slats (NaF, KF, LiF). Several inorganic materials were synthesized within molten salts, encompassing binary oxides,^[^ ^20^, ^21^, ^22^ ^]^ ternary oxides,^[^ ^23^, ^24^, ^25^ ^]^ as well as borides,^[^ ^26^ ^]^ and porous nitrides.^[^ ^27^ ^]^ Talapin et al. used molten salts as reaction media for cation exchange reactions on InP and InAs quantum dots.^[^ ^28^, ^29^ ^]^ In addition, GaN and AlN nanocrystals were directly synthesized from their halide salts under an ammonia atmosphere in a biphase mixture of organic surfactants and molten halide salt.^[^ ^30^ ^]^ Finally, Kan et al. used a eutectic mixture of MgCl_2_ and NaCl as a reaction medium for synthesizing TiN from TiO_2_ and Mg powder in a nitrogen (N_2_) atmosphere.^[^ ^31^ ^]^ Broad peaks pertaining to TiN were detected in XRD from 600 °C.^[^ ^32^ ^]^

## 2 Results and Discussion

**Figure 1:**
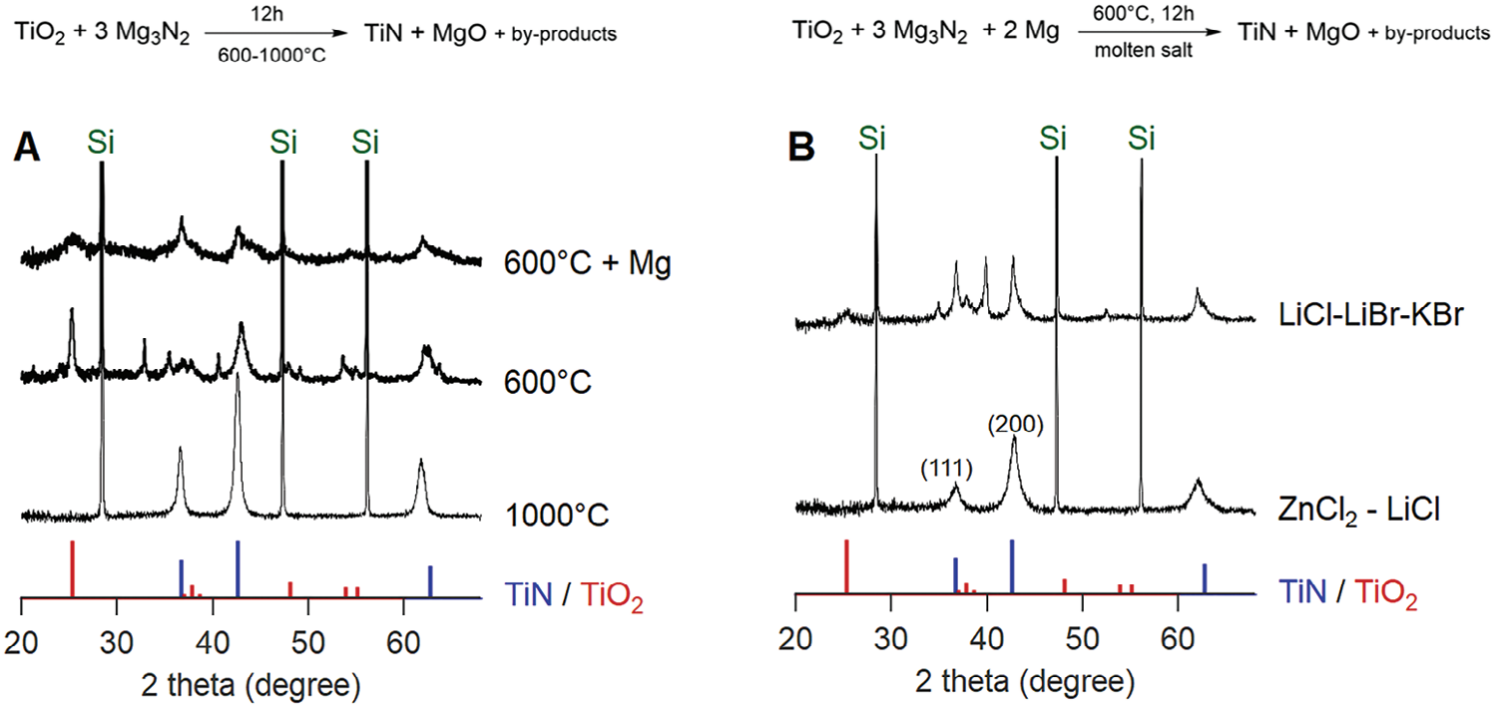
A) XRD patterns of solid state metatheses at 1000, 600, or at 600 °C  with the addition of two equivalents Mg. B) XRD patterns of syntheses in different molten salts (1.25 mmol of TiO_2_ in 544 mg of salt, yielding a titanium molality of 2.3 mol kg^–1^) with additional Mg. References of TiN (ICSD 26947), TiO_2_ (ICSD 9161), and silicon are indicated in blue, red, and green, respectively. The diffraction patterns were normalized to the internal silicon reference.

Here, we take the metathesis reaction and introduce molten salts as the reaction medium and Mg as the reducing agent, thus achieving the synthesis of TiN at temperatures as low as 350 °C and a significantly shortened reaction time of 1 hour. We first lowered the reaction temperature to 600 °C  in the regular solid‐state metathesis. By X‐ray diffraction (XRD), we mostly detected the reflections of titanium oxide (**Figure** **1**A), although some broad and shifted reflections reminiscent of oxygenated TiN were present as well. When magnesium is added as a reductant at 600 °C, all reflections become broader.

To remove activation barriers and provide faster diffusion, we explored several molten salts (see Table S1, Supporting Information, for their composition, and melting and boiling points). We avoided salts with oxygen to avoid any titanium oxide formation and favored salts with a low melting point (below 350 °C). Aluminum (AlCl_3_–NaCl–KCl) and thiocyanate (KSCN–NaSCN) eutectic salts were found to decompose at the reaction temperature and were also discarded due to their low boiling points. Products could be isolated from the alkali halide mixture (LiCl–LiBr–KBr) and from the ZnCl_2_–LiCl mixture. As depicted in Figure 1B, the alkali halide salts yield a side product while the ZnCl_2_–LiCl mixture yielded phase pure TiN, albeit with broadened reflections at 2θ angles of 36°, 42°, 61°, 74°, and 78° in the diffractogram. This outcome may be attributed to the Lewis acidity of ZnCl_2_, but also to the lower concentration of lithium in the molten salt. Indeed, titanium oxide is being explored as an anode material in lithium‐ion batteries, because of its ability to incorporate lithium.^[^ ^33^ ^]^ In the ZnCl_2_–LiCl mixture, there is 22 mol% of lithium (0.8 equivalents with respect to TiO_2_) while there is 62 mol% in the alkali halide mixture (14.0 equivalents) see Table S1 (Supporting Information). We varied the concentration of titanium oxide in the molten salt by changing the molar ratio of TiO_2_ to ZnCl_2_ from 1 to 12 equivalents, see Figure S2 (Supporting Information). A very high concentration is achieved by using only 1 equivalent of ZnCl_2_ (titanium molality of 6.8 mol kg^–1^ salt) and this does not result in complete TiN formation. A 1:3 molar ratio of TiO_2_ versus ZnCl_2_ (titanium molality of 2.3 mol kg^–1^) produces phase pure TiN. Too diluted concentrations, i.e., 12 equivalents (titanium molality of 0.57 mol kg^–1^) resulted in mostly TiN but a side product is also present.

**Figure 2:**
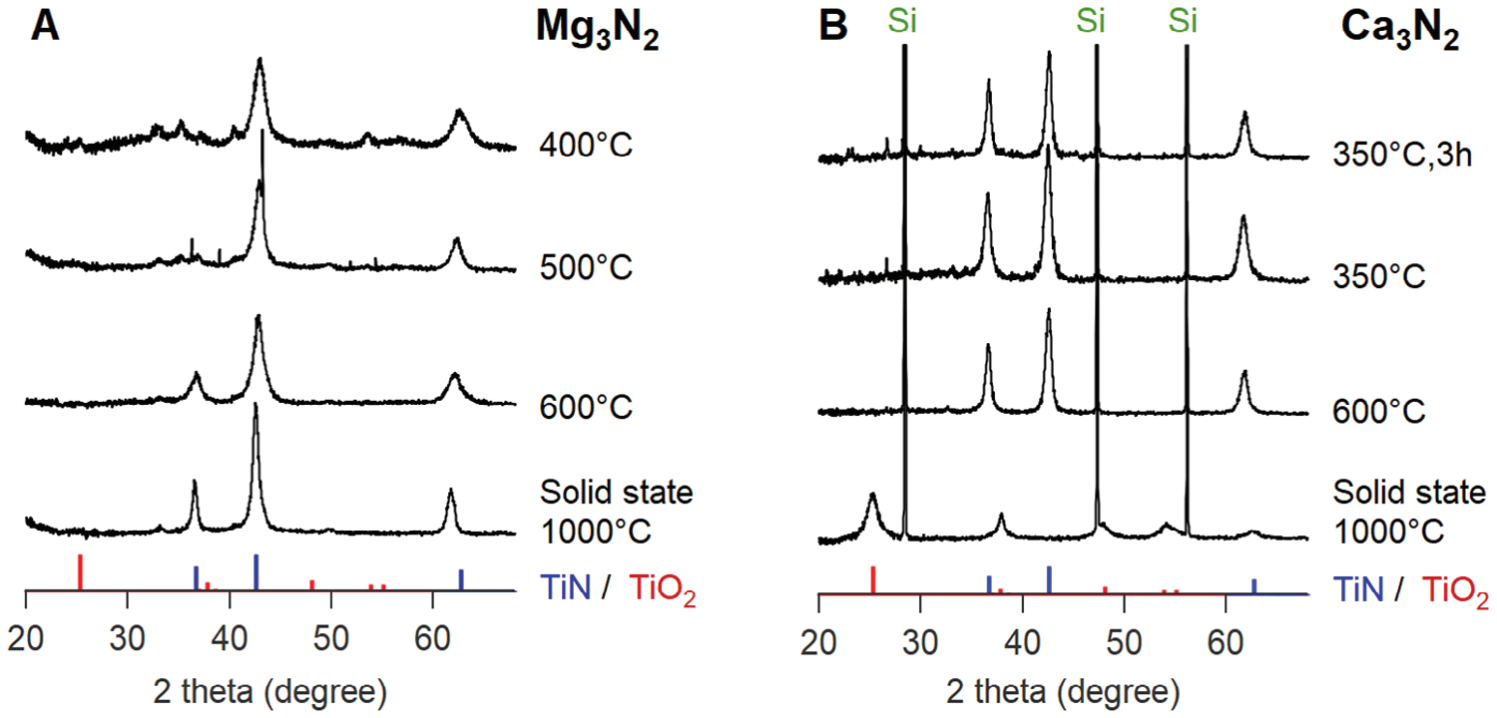
XRD patterns for 12 or 3 h reactions of TiO_2_ with A) Mg_3_N_2_ or B) Ca_3_N_2_ at various temperatures in ZnCl_2_–LiCl molten salt (3 equivalents salt). Two equivalents of magnesium were added in each case, except for the solid‐state metathesis reactions at 1000 °C. References of TiN, TiO_2_, and silicon are indicated in blue, red, and green, respectively. The diffraction patterns were normalized to an internal silicon reference for the reactions with Ca_3_N_2_.

Various alternative nitrogen sources were explored, including Li_3_N, NaNH_2_, and Ca_3_N_2_. While phase‐pure TiN was not obtained with Li_3_N and NaNH_2_ (see Figure S3, Supporting Information), Ca_3_N_2_ gave phase pure TiN at 600 °C  in the ZnCl_2_–LiCl molten salt, see **Figure** **2**. The TiN reflections are highly similar to the ones of the solid‐state reference with Mg_3_N_2_ at 1000 °C, see Figure 2. This is remarkable since the molten salt reaction with Mg and Mg_3_N_2_ at 600 °C, resulted in broader reflections. Scherrer analysis of the XRD pattern for the sample obtained via Ca_3_N_2_ reveals a crystallite size of 10.5 ± 0.4 nm, whereas TiN obtained via Mg_3_N_2_ shows a size of 4.6 ± 1 nm. Lowering the temperature further is not possible with Mg_3_N_2_ as TiN is no longer observed. With Ca_3_N_2_, we can obtain TiN at 350 °C, and we can even reduce the reaction time from 12 to 3 h. On the other hand, Ca_3_N_2_ does not produce TiN when used in the solid‐state metathesis at 1000 °C (without Mg), see Figure 2. Efforts to generate larger TiN NCs from larger TiO_2_ particles have been documented in the literature, however, all yielding similar sizes of TiN.^[^ ^2^ ^]^

At 350 °C, we explored the influence of various parameters (see Figure S4, Supporting Information). Five equivalents of molten salt (instead of three equivalents) led to the formation of side phases. Five equivalents of Ca_3_N_2_ (instead of three equivalents) yielded an identical result, while the addition of Mg_3_N_2_ again led to side phases and even remaining TiO_2_. A reaction performed in the absence of LiCl (but keeping a titanium molality of 2.3 mol kg^–1^) in ZnCl_2_ yielded similar results to those obtained with LiCl (Figure S5, Supporting Information), while the reaction without Mg resulted in an incomplete reaction and in the presence of a side phase (Figure S6, Supporting Information). Until here, the reactions were done in an alumina crucible inside a tube furnace. Due to the significant reduction in reaction temperature, we were able to conduct the synthesis using standard borosilicate glassware at the Schlenk line, leading to identical results, see Figure S7 (Supporting Information). During the reaction conducted in the flask, an abrupt autoignition event occurred at approximately 230°C, likely indicating the start of nitride formation. Subsequent analysis revealed that an optimal reaction duration of 1 h post‐autoignition led to the production of phase‐pure TiN. Shorter reaction periods (15 min post‐autoignition) resulted in the formation of a mixture comprising TiN product alongside unreacted titania, see Figure S8 (Supporting Information).

The differences between Ca_3_N_2_ and Mg_3_N_2_ are striking but not unprecedented.^[^ ^34^, ^35^ ^]^ In the metathesis of TiCl_3_ with Ca_3_N_2_ and Mg_3_N_2_ to TiN, it was also reported that Ca_3_N_2_ has a higher reactivity, which is related to a higher exothermic character of the reaction.^[^ ^36^ ^]^ For Equation 1, we calculate the enthalpy of the reaction to be Δ*H* = –294 kJ mol^–1^ TiO_2_. The equivalent reaction with Ca_3_N_2_ is also thermodynamically more favorable, with Δ*H* = –381 kJ mol^–1^ TiO_2_.^[^ ^37^, ^38^ ^]^ However, Ca_3_N_2_ does not produce TiN in the solid‐state reaction, which we relate rather to kinetic factors. The melting point of Ca_3_N_2_ is 1195 °C while Mg_3_N_2_ has a formal melting point of 800 °C, but already decomposes around 300 °C.^[^ ^39^, ^40^ ^]^ This makes the reaction with Ca_3_N_2_ a true solid‐state reaction with slow kinetics while the reaction with Mg_3_N_2_ proceeds in a melt, with (potentially more reactive) decomposition products of Mg_3_N_2_. Finally, the reaction with added magnesium as a reductant is indeed more exothermic than reaction 1, see Equations (2) and (3).

(2) $$\begin{array}{ccc} 2\;{\mathrm{TiO}}_{2}+{\mathrm{Mg}}_{3}N_{2}+\mathrm{Mg} & & ⟶2\;\mathrm{TiN}+4\;\mathrm{MgO}\\ \;\Delta H & & =-371\;kJ/mol\;{\mathrm{TiO}}_{2} \end{array}$$

(3) $$\begin{array}{ccc} 2\;{\mathrm{TiO}}_{2}+{\mathrm{Ca}}_{3}N_{2}+\mathrm{Mg} & & ⟶2\;\mathrm{TiN}+3\;\mathrm{CaO}+\mathrm{MgO}\\ \;\Delta H & & =-436\;kJ/mol\;{\mathrm{TiO}}_{2} \end{array}$$

Attempts to synthesize HfN and ZrN from oxide particles at 350°C in ZnCl_2_‐LiCl molten salts in the presence of Mg powder did not result in the desired nitride formation. Thermodynamic calculations indicate that ZrN formation (–305 kJ mol^–1^) is indeed less favorable than TiN (–436 kJ mol^–1^). Further investigation is necessary in this matter.

**Figure 3:**
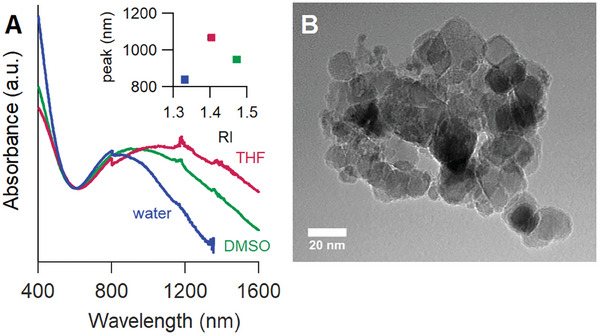
A) UV–vis absorption spectra of the TiN particles dispersed in various solvents. Inset: the top of the LSPR band in function of the refractive index of each solvent and B) TEM image for TiN synthesized at 350 °C  through a standard 12 h molten salts‐assisted reaction.

All products were washed with acid to remove MgO and leftover reagents. The particle aggregates are stabilized in polar solvents via charge stabilization, evidenced by zeta potential measurements (Figure S9, Supporting Information). Attempts to de‐aggregate the particles using ligands such as oleic acid were not successful (Figure S9, Supporting Information). The aggregated particles are dispersed in water, displaying distinct plasmonic behavior, as indicated by the broad absorption spectra illustrated in **Figure** **3**A, consistent with previous reports in the literature.^[^ ^2^, ^4^, ^41^ ^]^ The absorption band exhibited a shift in other solvents with higher refractive indices, such as DMSO and THF, consistent with plasmonic behavior. The broad absorption is attributed to the considerable polydispersity in particle size, as indicated by transmission electron microscopy images, see Figure 3B. High resolution TEM images did demonstrate the crystalline nature of the material (Figure S12, Supporting Information).

Analysis using energy‐dispersive X‐ray spectroscopy (EDX) indicated the particles after washing with HCl have a metal content of 80 atom% titanium, 13 atom% magnesium, and 7 atom% zinc (see Figure S10, Supporting Information). X‐ray photoelectron spectroscopy (XPS) was further used to investigate the surface chemistry of samples washed with HCl and acetic acid (Figure S11, Supporting Information). After an HCl wash the survey spectra show considerable amounts of Zn and Mg (consistent with the EDX measurements), whilst the HAc‐washed samples do not show them in any appreciable quantities. The Ti 2*p* spectrum of the HAc‐washed sample shows two chemical environments, which can be assigned to nitride at 455.1 eV and surface oxide at 459.0 eV. The presence of these environments is further confirmed by the N and O 1*s* spectra. Whilst some signatures of both the nitride and oxide environments are also visible in the HCl‐washed sample, it is difficult to clearly assign all spectral features due to the presence of Mg and Zn salts and related differential charging of particles.

## 3 Conclusion

This study presents an unprecedented method for synthesizing TiN at low temperatures, rendering TiN, accessible under standard conditions using borosilicate glassware. Various innovations were essential: i) Selection of a molten salt that does not form side products (e.g., ZnCl_2_). ii) The use of magnesium as a reductant. iii) Identifying calcium nitride as a nitride source with a different temperature‐dependent reactivity than magnesium nitride. The latter two factors increase the thermodynamic driving force of the reaction. Titanium nitride nanoparticles might compete in the future with Au nanoparticles due to their comparable light absorption properties and the significantly lower cost of titanium.

## Conflict of Interest

The authors declare no conflict of interest.

## Supporting information

Supporting Information

## Data Availability

The data that support the findings of this study are openly available in Zenodo at https://doi.org/10.5281/zenodo.10551742 , reference number 10551742.
