# Supplementary material for: Molten Salt‐Assisted Synthesis of Titanium Nitride
Source: Small Methods. 2024 Jun 10;8(12):2400228. doi: 10.1002/smtd.202400228 (PMC11671857; doi:10.1002/smtd.202400228)
Supplement: Supplementary file 1 — Supporting Information [file SMTD-8-2400228-s001.pdf]

small methods

Supporting Information

for *Small Methods*, DOI 10.1002/smtd.202400228

Molten Salt-Assisted Synthesis of Titanium Nitride

*Mahsa Parvizian, Nico Reichholf, Aysha A. Riaz, Prajna Bhatt, Anna Regoutz and Jonathan De Roo**

Supporting Information: Molten Salt-Assisted Titanium Nitride Synthesis

Mahsa Parvizian,† Nico Reichholf,† Aysha A. Riaz,‡ Prajna Bhatt,‡ Anna Regoutz,‡ and Jonathan De Roo*,†

†*Department of Chemistry, University of Basel, Basel CH-4058, Switzerland*

‡*Department of Chemistry, University College London, 20 Gordon Street, London, WC1H 0AJ, United Kingdom.*

E-mail: Jonathan.DeRoo@unibas.ch

Experimental section

Materials

The precursors employed in the synthesis included commercially available titanium(IV) oxide nanopowder (TiO_2, 21 nm , ≥99.5% trace metals basis, Sigma), magnesium(II) nitride cubic phase (M_3N_2,-325 mesh, ≥99.5%, Sigma), calcium(II) nitride (Ca_3N_2,-200 mesh, 99% , Sigma), magnesium powder (Mg, ≥99%, Sigma), anhydrous zinc(II) chloride ($ZnCl_2$, ≥97%, Sigma), and lithium chloride (LiCl,99%, Strem). In other reported reactions, sodium amide ($NaNH_2$, 98%, Sigma), lithium nitride (Li_3N,-80 mesh, ≥99.5%, Sigma), lithium bromide anhydrous (LiBr,99+%, Strem), and potassium bromide (KBr, 99%, Sigma) were utilized. All precursors were stored within a nitrogen-filled glovebox to prevent exposure to moisture. Subsequently, for purification purposes, the following reagents were used: distilled water, absolute ethanol, acetone, chloroform, cyclohexane, acetic acid (CH_3CO_2H,¿99%, Sigma), and oleic acid ($C_{18}H_{34}O_2$, 90%, Sigma).

Syntheses without molten salts

This is the reference method by Karaballi *et al.*.[S1] In a nitrogen-filled glovebox, TiO_2 nano-powder (100 mg, 1.25 mmol, 1 eq.) was combined with Mg_3N_2 powder (378 mg, 3.75 mmol, 3 eq.). This mixture was ground using an agate mortar and pestle for 10 min, yielding a fine homogeneous powder. The resulting blend was then transferred to an aluminium oxide combustion boat and swiftly positioned inside a quartz tube within a Nabertherm N7/H tube furnace filled with argon. The mixture underwent heating at 1000 °C for 12 h (rate of 13 °C/minute) under an argon flow.

Following the reaction and once the furnace had cooled, the particles were dispersed in 15 mL of distilled water and stirred with a 20 mL 1M HCl solution for 1 h at room temperature (RT). Afterward, the mixture was centrifuged at 5000 rcf for 4 minutes. The precipitate was washed once more with 30 mL of distilled water. Finally, the particles were dispersed in 5 mL of distilled water.

Syntheses with molten salts

In a nitrogen-filled glovebox, TiO_2 nano-powder (100 mg, 1.25 mmol, 1 eq.) was combined with Mg_3N_2 (378 mg, 3.75 mmol, 3 eq.) or Ca_3N_2 (555 mg, 3.75 mmol, 3 eq.) with magnesium powder (60 mg, 2.50 mmol, 2 eq.). This mixture was blended with an eutectic combination of $ZnCl_2$ (500 mg, 3.66 mmol) and LiCl (44 mg, 1.03 mmol) using an agate mortar and pestle for 10 min, yielding a fine homogeneous powder. The resulting blend was then transferred to an aluminium oxide combustion boat and swiftly positioned inside a quartz tube within a Nabertherm N7/H tube furnace filled with argon. The mixture underwent heating at different temperatures ranging from 350–600 °C for 12 h (heating rate of 13 °C/minute) with an argon flow.

After the reaction, upon the cooling of the oven, the resulting powder was washed using water as a high dielectric constant polar solvent. Four rounds of centrifugation with 30 mL water at 5000 rcf for 4 min were necessary to eliminate most of the salts. Subsequently, the

particles were dispersed in 15 mL water and stirred with 20 mL of 1M HCl solution for 1 h at RT. Afterward, the mixture was centrifuged at 5000 rcf for 4 minutes. The precipitate was washed once more with 30 mL of distilled water. Finally, 100 mg of the product was obtained and dispersed in 5 mL of distilled water. This purification method was our main approach.

An alternative purification method was explored with carboxylic acids. In this method, the particles were dispersed in 40 mL of water and agitated with 3 mL of acetic acid at 50 °C for 48 h. After this acid treatment, the particles required four rounds of washing and centrifugation with water to eliminate the acetate side products. In each round, the particles are dispersed in 30 mL of water and centrifuged at 5000 rcf for 4 minutes. Visual differentiation between the nitride product and grey-colored acetate side products guided this step. Subsequently, the particles were stabilized using an organic ligand, oleic acid. The particles were once again dispersed in 40 mL of water, and mix with 2 mL of oleic acid and 20 mL of cyclohexane, stirred vigorously for 2 h at 50 °C. Subsequently, the particles were collected from the cyclohexane phase. If the phase separation was not distinctly evident, the mixture of water and cyclohexane underwent centrifugation at 500 rcf for 4 minutes, facilitating a more pronounced phase separation. The NCs were then precipitated with 40 mL of acetone and centrifugated at 5000 rcf for 4 minutes. The resulting precipitate was dispersed in 10 mL chloroform to which 200 L of oleic acid was added, followed by 15 minutes of sonication. The particles were precipitated using 30 mL of acetone and centrifuged at 5000 rcf for 4 minutes. This process of stabilization involving 200 L of oleic acid in chloroform, followed by acetone purification, was repeated. The final outcome was a suspension of TiN capped with oleic acid in 5 mL of chloroform.

Instrumentation

Powder x-ray diffraction (XRD) spectra were measured using a STOE StadiP powder diffractometer with a Dectris Mythen 1K detector and a micro-focused Cu Kα source (λ=1.542 Å).

In the XRD analysis, 4 mg of each purified product was weighed alongside 2 mg of silicon (33 m%), which served as an internal reference. The silicon's intensity was normalized, enabling a direct comparison of diffraction intensities among the products derived from various syntheses. TEM imaging was done using a JEOL JEM2800 field emission gun microscope operated at 200 kV equipped with a TVIPS XF416ES TEM camera. UV-vis spectra were recorded with a J&M TIDAS spectrometer.

X-ray photoelectron spectroscopy (XPS) was done using laboratory-based Thermo Scientific Nexsa and Kratos Supra spectrometers equipped with monochromated Al Kα sources (hv = 1486.6 eV). All spectra were collected with a flood gun to reduce excessive charging and with the maximum X-ray spot size of 400 m to optimise the efficiency of collecting high-resolution spectra. Pass energies of 20 and 200 eV were used for the collection of core level and survey spectra, respectively. Data processing of the core level and survey spectra was performed using the Thermo Avantage and CASA software packages.

Supplementary Results

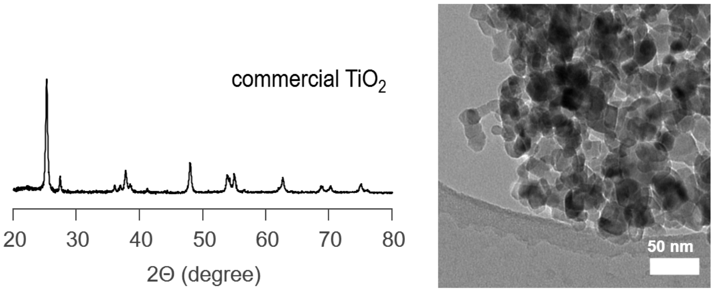

Figure S1: TEM and XRD analysis of the commercially available TiO2 precursor. The XRD reveals a mixture of rutile and anatase phases. Previous literature has addressed that altering the size of the precursor does not affect the final product's size; hence, investigations along that line were not pursued.

Table S1: The different sets of eutectic molten salts considered for our reaction with their corresponding melting points (*mp*) and boiling point (*bp*).

**Molten salts**	mol.%	*mp* (°C)	*bp* (°C)
LiCl/LiBr/KBr	25.0 : 37.0 : 38.0	320	>900
$AlCl_3$/NaCl/KCl	63.5 : 20.0 : 16.5	90	≈ 220
NaSCN/KSCN	26.3 : 73.7	140	≈ 500
$ZnCl_2$/LiCl	78.0 : 22.0	280	>900
$MgCl_2$/NaCl	43.0 : 57.0	459	>900

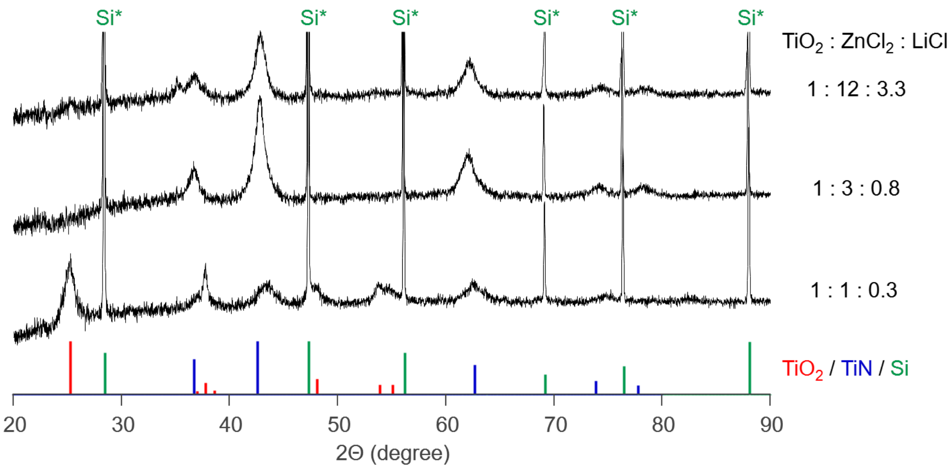

Figure S2: XRD patterns of products obtained by varying the $ZnCl_2$ to TiO_2 molar ratio at 600 °C for 12 h. Mg_3N_2 served as the nitrogen source, with Mg as the reducing agent. The eutectic molten salt mixture comprised 78 mol% $ZnCl_2$ and 22 mol% LiCl. References of TiN, TiO_2, and silicon are indicated in blue, red, and green, respectively. The diffraction patterns were normalized to the internal silicon reference.

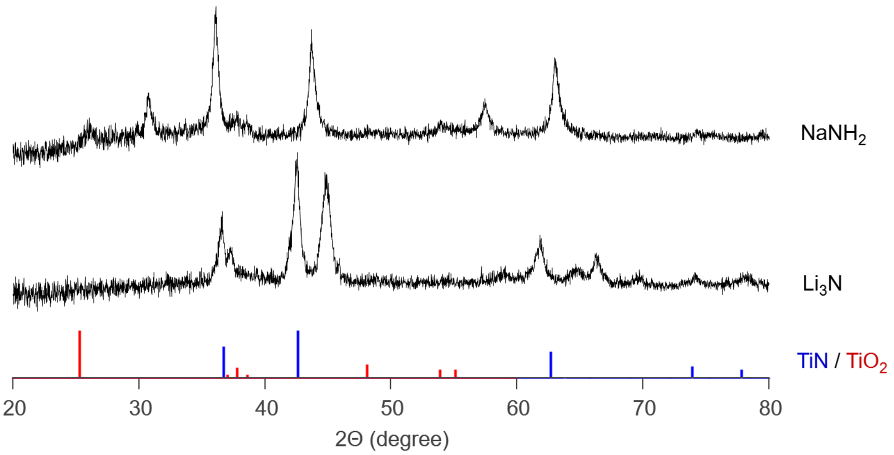

Figure S3: XRD pattern of products obtained after reaction with $NaNH_2$ and Li_3N at 600 °C for 12 h in molten salts. Both nitrogen sources did not yield phase pure TiN. References of TiN and TiO_2 are indicated in blue and red respectively.

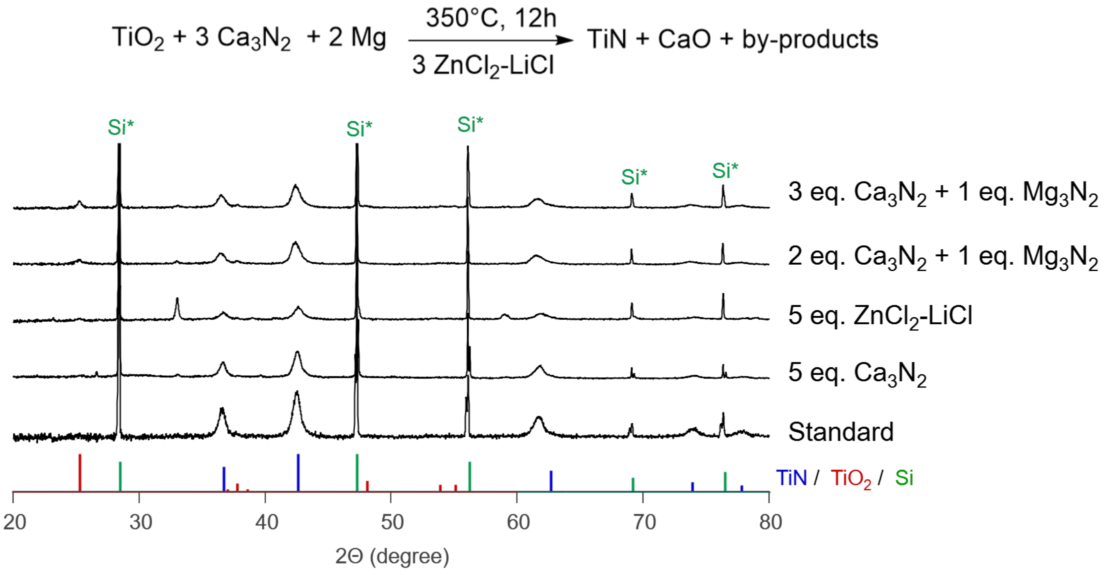

Figure S4: XRD pattern of products obtained after various variations at 350 °C for 12 h in $ZnCl_2$-LiCl molten salts. The diffraction patterns were normalized to the internal silicon reference.

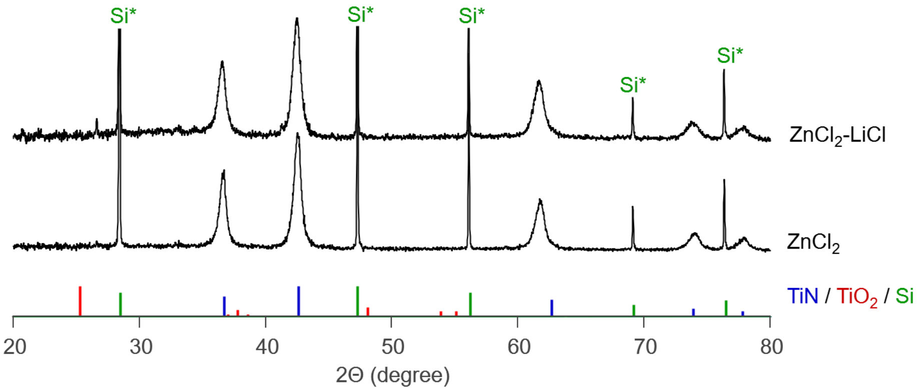

Figure S5: XRD patterns of products obtained at 350 °C for 12h with and without LiCl with titanium molality of 2.3 mol/Kg and 2 eq. of magnesium. References of TiN, TiO_2, and silicon are indicated in blue, red, and green, respectively. The diffraction patterns were normalized to the internal silicon reference.

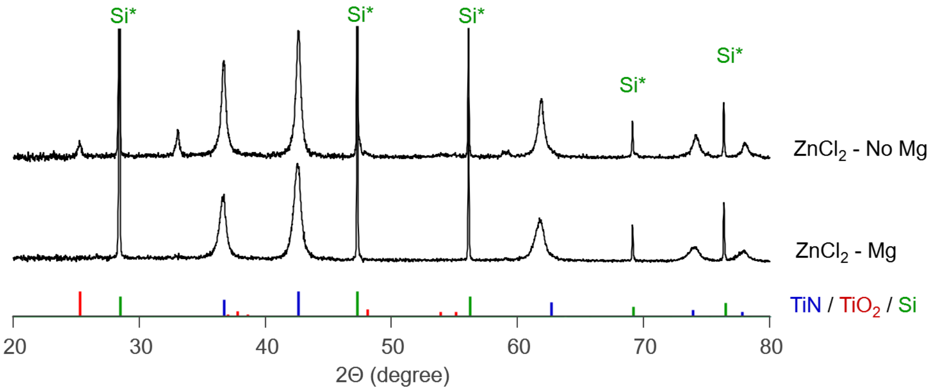

Figure S6: XRD patterns of products obtained after a 3h reaction at 350 °C in $ZnCl_2$ molten salt with a titanium molality of 2.3 mol/Kg and with or without 2 equivalents of magnesium. References of TiN, TiO_2, and silicon are indicated in blue, red, and green, respectively. The diffraction patterns were normalized to the internal silicon reference. The reaction without Mg resulted in the formation of an unknown side phase.

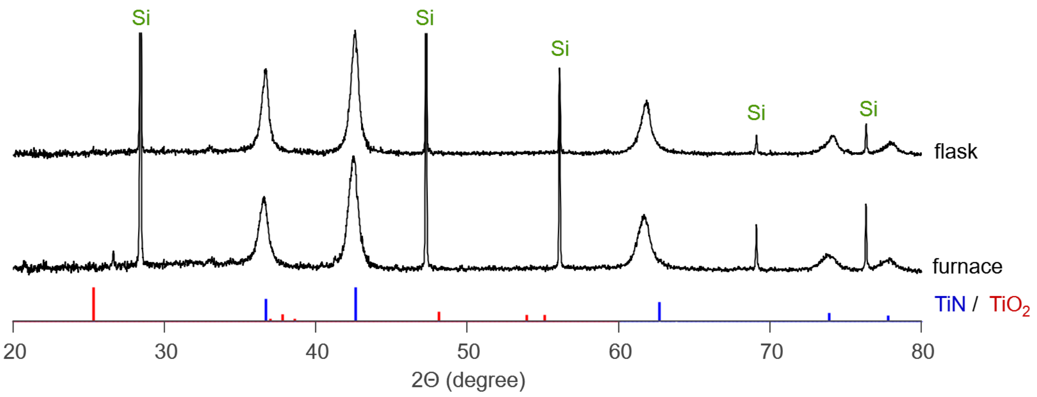

Figure S7: XRD patterns of products obtained in 3-neck flask setup after a 12h reaction at 350 °C in $ZnCl_2$ molten salt with a titanium molality of 2.3 mol/Kg and 2 equivalents of magnesium. References of TiN, TiO_2, and silicon are indicated in blue, red, and green, respectively. The diffraction patterns were normalized to the internal silicon reference.

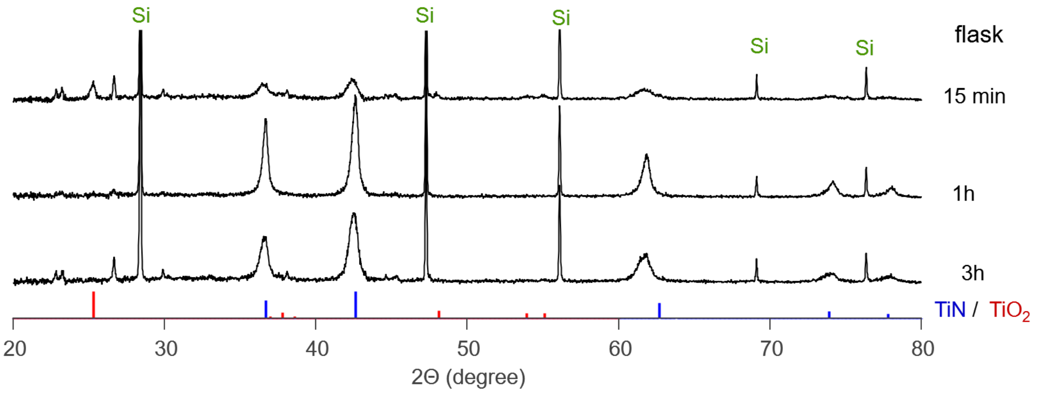

Figure S8: XRD patterns of products obtained in 3-neck flask setup after 3 h, 1 h, and 15 min reactions at 350 °C in $ZnCl_2$ molten salt with a titanium molality of 2.3 mol/Kg and 2 equivalents of magnesium. References of TiN, TiO_2, and silicon are indicated in blue, red, and green, respectively. The diffraction patterns were normalized to the internal silicon reference.

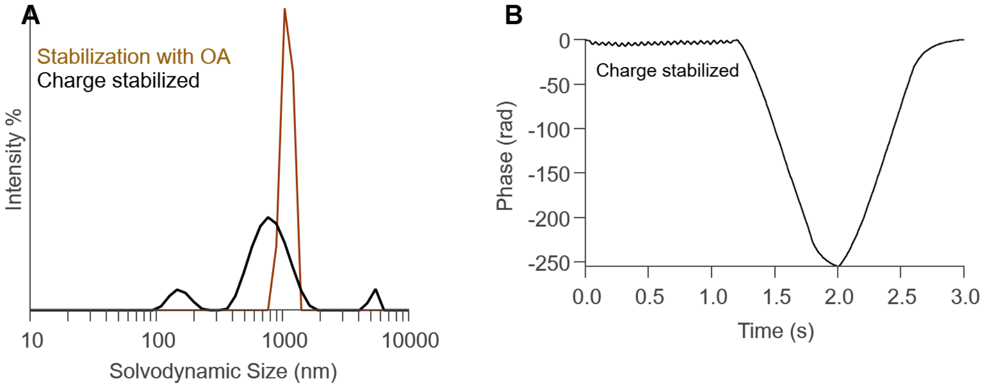

Figure S9: DLS measurement comparison of TiN particles synthesized at 350°Cfor 12 h, post-water and HCl purification and charge stabilization, against oleic acid-capped particles, revealing instability and aggregation in both cases. Oleic acid stabilization is observed to stabilize aggregates rather than individual particles(A). Zeta potential data of the same charge stabilized particles indicates a negatively charged surface (-24.04 mV) (B).

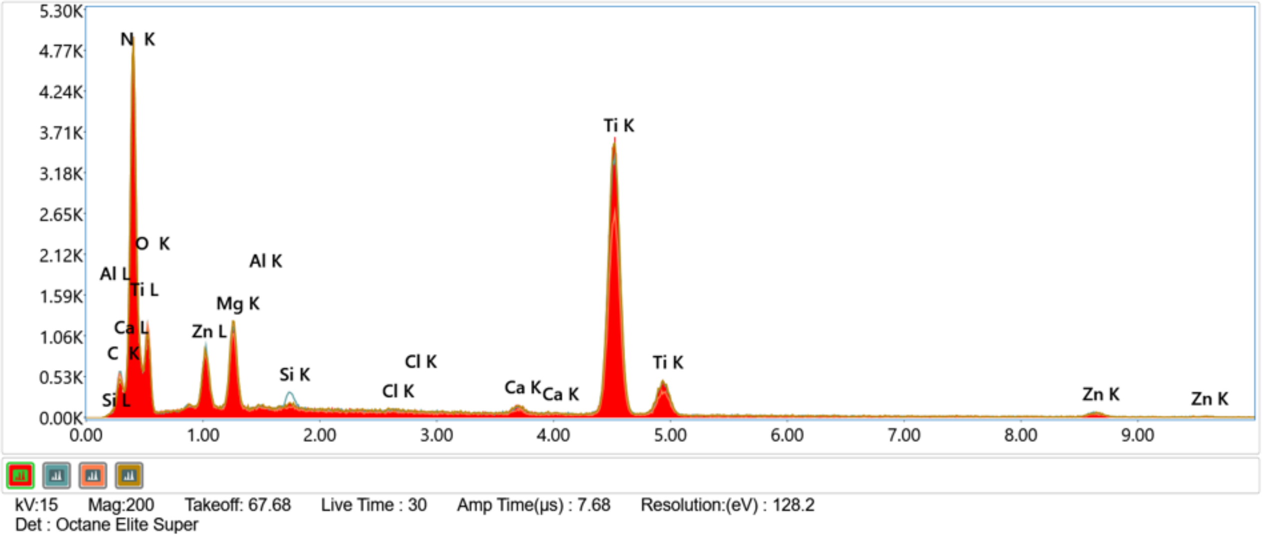

Figure S10: SEM EDX analysis of TiN particles synthesized at 350°C for 12 h, post-water and HCl purification and charge stabilization.

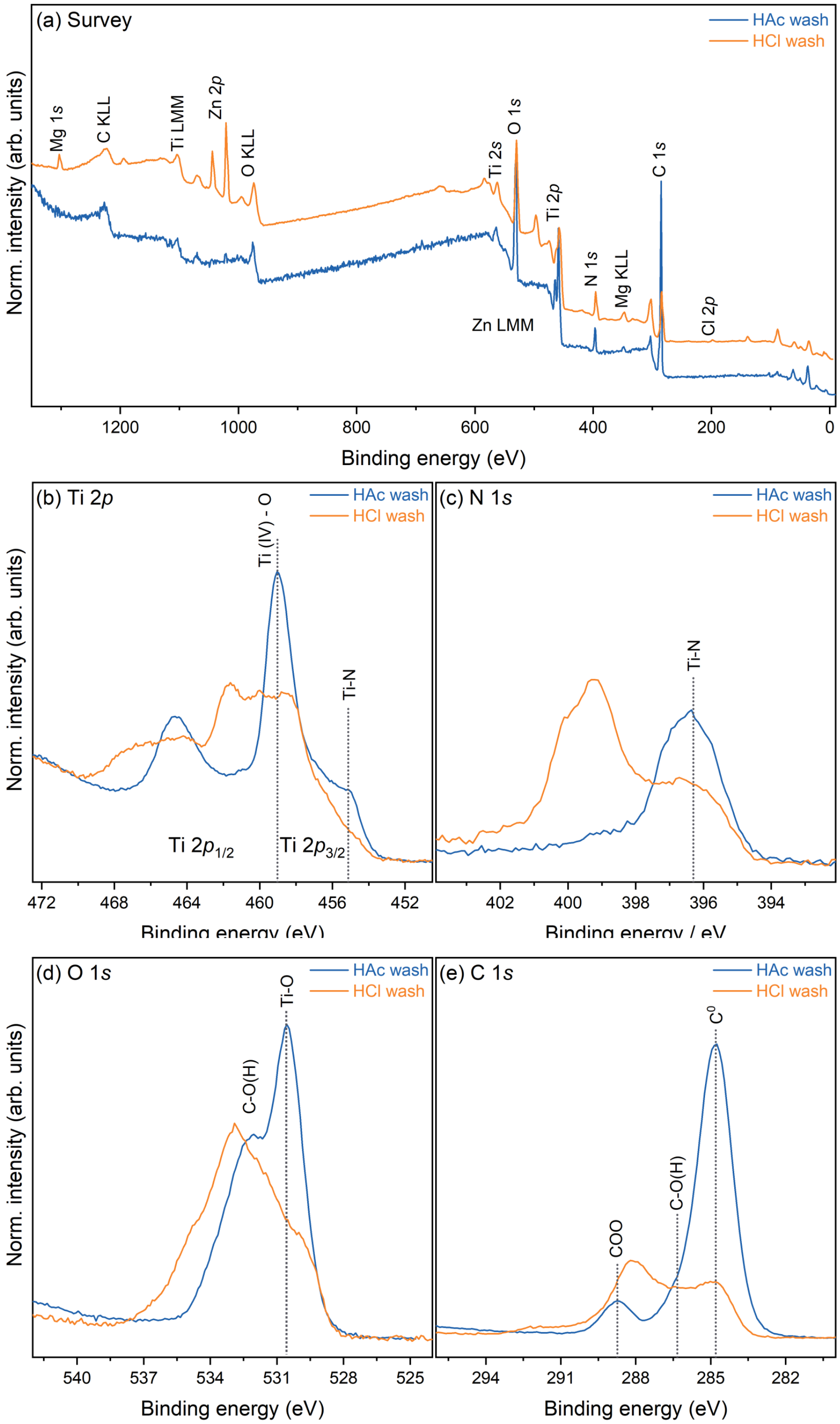

Figure S11: XPS analysis of TiN particles synthesized at 350°C for 12 h, post-water purification and either HCl or acetic acid (HAc) wash. All core level spectra are normalised to the total Ti 2p area.

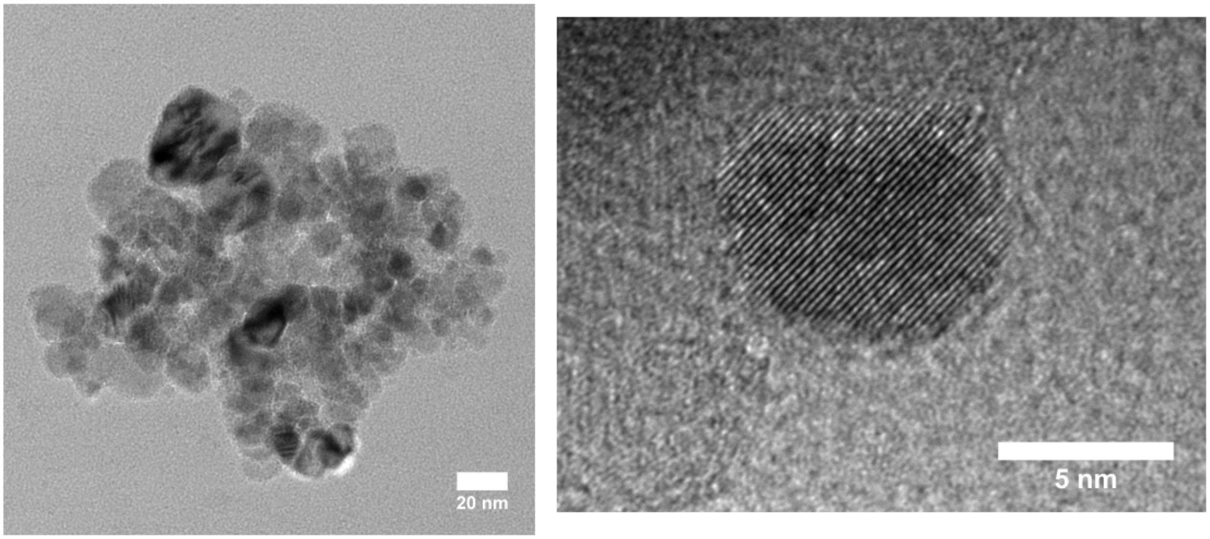

Figure S12: TEM analysis of TiN particles synthesized at 350°C for 12 h, post-water and HCl purification and charge stabilization.

References

(S1) Karaballi, R. A.; Humagain, G.; Fleischman, B. R. A.; Dasog, M. Synthesis of Plasmonic Group-4 Nitride Nanocrystals by Solid-State Metathesis. *Angewandte Chemie International Edition* **2019**, *58*, 3147–3150.
